# Efficacy and Safety of Ultrasound Guided-Deep Serratus Anterior Plane Blockade With Different Doses of Dexmedetomidine for Women Undergoing Modified Radical Mastectomy: A Randomized Controlled Trial

**DOI:** 10.3389/fmed.2022.819239

**Published:** 2022-02-07

**Authors:** Xia Xu, Xingfang Chen, Wenchao Zhu, Jing Zhao, Yanchao Liu, Caiping Duan, Yingying Qi

**Affiliations:** ^1^Department of Anesthesiology, Liaocheng People's Hospital, Liaocheng, China; ^2^Department of Anesthesiology, Ordos Central Hospital, Ordos, China

**Keywords:** serratus anterior plane block, modified radical mastectomy, ultrasound, dexmedetomidine, ropivacaine

## Abstract

**Background:**

Ultrasound guided-deep serratus anterior plane block (USG-DSAPB) has been used for pain management of patients undergoing modified radical mastectomy (MRM), but evidence supporting their adjuvant analgesic benefits is limited. We explored the efficacy and safety of preemptive use of ropivacaine combined with different doses of dexmedetomidine (DEX) in USG-DSAPB for patients undergoing MRM.

**Methods:**

Ninety-five female patients undergoing unilateral MRM were allocated randomly to two groups. Group RD1 had 20 mL of 0.5% ropivacaine with 5 mg of dexamethasone and 0.5 μg·kg^−1^ DEX in USG-DSAPB. Group RD2 had 20 mL of 0.5% ropivacaine with 5 mg of dexamethasone and 1 μg·kg^−1^ DEX in USG-DSAPB. The primary outcome was sufentanil consumption 72 h after USG-DSAPB. Secondary outcomes were: postoperative pain scores and level of sedation; intraoperative hemodynamics; duration of post-anesthesia care unit (PACU) stay; prevalence of moderate-to-severe pain; one-time puncture success; procedure time of blockade; time to first rescue analgesia; requirement of rescue analgesia; satisfaction scores of patients and surgeons; duration of hospital stay; adverse events; prevalence of chronic pain; quality of postoperative functional recovery.

**Results:**

Compared with the RD1 group, the visual analog scale score for coughing was significantly lower at 4, 8, 12 h and sufentanil consumption was significantly lower at 4, 8, 12, 24, and 48 h after surgery in the RD2 group (*P* < 0.05). The time to first rescue analgesia was significantly longer in the RD2 group (*P* < 0.05). The requirement for rescue analgesia was significantly higher in the RD1 group (*P* < 0.05). The prevalence of moderate-to-severe pain, number of patients using vasoactive agents, duration of PACU stay, as well as consumption of propofol, remifentanil, and DEX were significantly lower in the RD2 group (*P* < 0.05). There were no significant differences between the two groups with respect to one-time puncture success, procedure time of blockade, total dermatomal spread, satisfaction scores of patients and surgeons, postoperative complications, duration of hospital stay, 40-item Quality of Recovery questionnaire (QoR-40) score, or prevalence of chronic pain (*P* > 0.05).

**Conclusions:**

We discovered that 1 μg·kg^−1^ (not 0.5 μg·kg^−1^) DEX combined with 20 mL of 0.5% ropivacaine and 5 mg of dexamethasone in USG-DSAPB could provide superior postoperative analgesia for patients undergoing MRM. However, the quality of postoperative functional recovery and prevalence of chronic pain were similar.

**Clinical Trial Registration:** http://www.chictr.org.cn/showproj.aspx?proj=54929, identifier: ChiCTR2000033685.

## Introduction

Breast cancer is the most common cancer in women worldwide. It accounts for nearly one-third of all new cancer cases in women (1). Modified radical mastectomy (MRM) is first-line treatment for early, localized breast cancer (though use of chemotherapy and endocrine therapy before surgery is increasing) (2). Scholars have reported that >35% of breast-cancer patients suffer acute pain following MRM even though surgical methods tend to be minimally invasive (3). Postoperative acute pain is a high risk factor for the development of postmastectomy pain syndrome, which can impair quality of life (4, 5). One of the most important reasons for postoperative pain is the scant attention paid to its management compared with that after other types of cancer surgery (6).

In recent years, regional anesthesia has frequently been preferred as part of multimodal analgesia because it is more effective and associated with fewer side-effects (7–10). Thoracic epidural analgesia, intercostal nerve blockade, paravertebral blockade, and local infiltration are common methods of analgesia after MRM. However, each method has advantages and disadvantages (11–14). As a result, less invasive strategies for regional analgesia are being investigated. Recently, thoracic plane blockade has been proposed as a novel and rapidly expanding facet of regional anesthesia. In particular, ultrasound guidance has been introduced to improve the success and safety of regional nerve blockade (15, 16).

Ultrasound guided-serratus anterior plane blockade (USG-SAPB) was first defined by Blanco in 2013. It has been used for pain control after breast surgery because of the excellent analgesia it induces, lower invasiveness, simplicity, ease of learning, and relative safety (17). It can block the lateral branches of the intercostal nerves of the T2–T9 spinal nerves by injecting the local anesthetic into the plane either superficial or deep to the serratus anterior muscle (18). Ultrasound guided-deep serratus anterior plane blockade (USG-DSAPB) has been used for the insertion of chest drains, reconstructive breast surgery, cosmetic breast surgery, and video-assisted thoracoscopic surgery (19). Compared with other types of thoracic plane blockade (e.g., interpectoral plane blockade, pectoserratus plane blockade), the local anesthetic is injected to a more dorsal side in the SAPB. As a result, USG-DSAPB can target the thoracic nerves more selectively and anesthetize more intercostal nerves (20). Local anesthetic combined with dexmedetomidine (DEX) has been reported to prolong analgesia in brachial plexus blockade (21). We explored the efficacy and safety of preemptive use of different concentrations of DEX and ropivacaine in USG-DSAPB for patients undergoing MRM.

## Methods

### Ethical Approval of the Study Protocol

The study protocol was approved by the Ethics Review Boards of Liaocheng People's Hospital (2014001; 17 January 2014; Liaocheng, China) and Ordos Central Hospital (2020-004; 8 June 2020; Ordos, China). It is registered at Chinese Clinical Trial Registry (ChiCTR2000033685). Written informed consent was obtained from patients before participation in this study. This study has been reported according to the CONSORT 2010 statement.

### Inclusion Criteria

The inclusion criteria were patients: (i) with American Society of Anesthesiologists (ASA) grade I–II; (ii) aged 45–60 years; (iii) scheduled for unilateral MRM with dissection of axillary lymph nodes; (iv) receiving patient-controlled intravenous analgesia (PCIA).

### Exclusion Criteria

The exclusion criteria were patients: (i) with contraindications to DSAPB (anticoagulant treatment or coagulative abnormality, infection at injection site, severe deformities in the chest wall); (ii) with known allergies to the drugs used in our study; (iii) who underwent radiotherapy before surgery; (iv) who had undergone secondary surgery; were heavy users of tobacco or drugs; had a history of motion sickness, peripheral (e.g., diabetic) neuropathy, severe cardiopulmonary disease, renal/liver dysfunction, or chronic pain; (v) unable to cooperate or communicate; (vi) had body mass index >30 kg/m^2^.

### Randomization and Blinding

Patients who underwent breast-cancer surgery between June and September 2020 were recruited. Patients were allocated randomly into two groups by a computer-generated random number list. Group RD1 had 20 mL of 0.5% ropivacaine with 5 mg of dexamethasone and 0.5 μg·kg^−1^ DEX in USG-DSAPB. Group RD2 had 20 mL of 0.5% ropivacaine with 5 mg of dexamethasone and 1 μg·kg^−1^ DEX in USG-DSAPB. Participants were unaware of the group assignment. Nurses in the Acute Pain Service Team educated patients on how to use a visual analog scale (VAS) and PCIA pump, prepared the study drugs, and carried out postoperative assessments.

### USG-DSAPB

Premedication was not given before USG-DSAPB. Patients underwent standard monitoring (non-invasive measurement of blood pressure, pulse oximetry, electrocardiography, temperature) according to ASA guidelines after arriving at the anesthesia preparation room. Venous access was established at the contralateral upper limb. The Bispectral Index (BIS) monitor was placed at the side of the forehead according to manufacturer instructions. Patients were sedated, analgesia instituted [midazolam (0.02 mg·kg^−1^) and fentanyl (1 μg·kg^−1^)] and oxygen (2 L/min) given after insertion of a nasal cannula.

The same anesthesiologist in each center, unaware of the group assignment, undertook DSAPB under real-time ultrasound guidance according to a method described previously (22). Briefly, patients were placed in the lateral decubitus position with the operation side up. After routine disinfection, a high-frequency linear probe (SonoSite, Bothell, WA, USA) was placed over the middle clavicular region in the sagittal plane. After subcutaneous tissue, latissimus dorsi, serratus anterior, intercostal muscles, and pleura had been identified at the fourth rib in the midaxillary line, a 22-G needle (B. Braun, Melsungen, Germany) was introduced from caudad-to-cephalad using plane technology. The needle location was confirmed with 2 mL of physiologic (0.9%) saline solution and an absence of blood or air upon aspiration. Then, 20 mL of 0.5% ropivacaine with 5 mg of dexamethasone and 0.5 or 1 μg·kg^−1^ of DEX was injected in the deep layer of the serratus anterior plane (between the serratus anterior muscle and external intercostal muscles) at the fourth thoracic vertebra for ~10 s. We defined “successful blockade” as a loss of cold sensation in >2 dermatomes 30 min after blockade followed by transfer to the operating theater. Otherwise, blockade was considered to have failed.

### Anesthesia

Anesthesia was induced by lidocaine (1.5 mg·kg^−1^) dexamethasone (0.1 mg·kg^−1^), fentanyl (2–3 μg·kg^−1^), propofol (1–2 mg·kg^−1^), and cisatracurium (0.1 mg·kg^−1^). A laryngeal mask was placed to control the airway intraoperatively. DEX (0.2–0.7 μg·kg^−1^·h^−1^), propofol (3–6 μg·mL^−1^), and remifentanil (0.05–0.2 μg·kg^−1^·min^−1^) were titrated to maintain the BIS at 50–60 and hemodynamics within 20% of baseline. Cisatracurium (0.05 mg·kg^−1^) was given at the discretion of the anesthetist. Tropisetron (5 mg) and ketorolac (30 mg) were administered (i.v.) ~30 min before the end of the surgical procedure. Neuromuscular blockade was reversed by neostigmine (0.02 mg·kg^−1^) and atropine (0.01 mg·kg^−1^), if necessary. Ten milliliters of 2% lidocaine was infiltrated (s.c.) in parasternal and subclavicular areas for postoperative analgesia by the surgeon according to a method described previously (23). The laryngeal mask was removed after the patient responded promptly to a command, and they were moved to the post-anesthesia care unit (PACU). MRM was undertaken using the same method by the same surgical team in each center according to a method described previously (24).

### Postoperative Pain Management

Patients received the same protocol for postoperative analgesia in both groups. At the end of the surgical procedure, PCIA was started with sufentanil (0.8 μg·mL^−1^). The bolus volume was 2 mL, background dose was 1 mL·h^−1^, locked time was 5 min, and 1-h limit was 12 ml. Flurbiprofen axetil (50 mg, i.v.) was administered every 8 h on the ward as part of multimodal analgesia management. If the VAS at rest was >3 after bolus administration of analgesics, ketorolac (30 mg, i.v.) was given. Postoperative nausea and vomiting (PONV) was treated with tropisetron (5 mg).

### Data Collection

The primary outcome was sufentanil consumption 72 h after surgery. The secondary outcomes were postoperative pain scores (VAS: 0 cm = no pain, 10 cm = worst pain imaginable) and level of sedation (LOS; 0 = alert; 1 = mildly drowsy; 2 = moderately drowsy, easily arousable; 3 = very drowsy, arousable; 4 = difficult to arouse; 5 = unarousable) recorded at 1, 2, 4, 8, 12, 24, 48, and 72 h postoperatively. The other secondary outcomes were intraoperative hemodynamics, duration of PACU stay, PONV (0 = no nausea; 1 = mild nausea; 2 = severe nausea; 3 = one episode of vomiting; 4 = vomiting more than once), prevalence of moderate-to-severe pain (VAS at rest >3), one-time puncture success, procedure time of blockade, time to first rescue analgesia, requirement of rescue analgesia, satisfaction scores of patients and surgeons (1 = extremely unsatisfied; 5 = extremely satisfied), duration of hospital stay, adverse events, and the prevalence of chronic pain 3 months after surgery. The quality of postoperative functional recovery was graded using the 40-item Quality of Recovery questionnaire (QoR-40; 40–200) and assessed the day before surgery, the first after day surgery, upon hospital discharge, and 3 months after surgery.

The mean arterial pressure (MAP) and heart rate (HR) were recorded upon arrival at the operating theater (T0), before DSAPB (T1), after DSAPB T2), before anesthesia induction (T3), after anesthesia induction (T4), before skin incision (T5), immediately after skin incision (T6), and extubation (T7). “Hypotension” was defined as MAP reduction >20% compared with that at baseline, and was treated with phenylephrine (40 μg) or ephedrine (6 mg). “Bradycardia” was defined as HR <60 bpm or reduction >20% compared with that at baseline, and treated with atropine (0.2 mg).

### Statistical Analyses

The sample size was calculated on the basis of a 15% reduction in the cumulative amount of sufentanil 72 h after the surgical procedure in our preliminary trial (85.92 ± 24.08 μg in the RD1 group). For a statistical power of 80% (α = 0.05, β = 0.2), 42 participants were required in each group according to PASS 11.0 (NCSS Statistical Software, Kaysville, UT, USA). Assuming a dropout percentage of 15%, the final sample size should be 49 patients in each group.

The Kolmogorov–Smirnov test was employed to assess the distribution of variables. The homogeneity of variance was determined using the Levene test. Quantitative data are expressed as the mean ± SD or median and interquartile range. Differences between groups were compared using repeated-measures analysis of variance with the Bonferroni correction for data with a normal distribution. For data with a non-normal distribution, groups were compared using the Mann–Whitney *U*-test. Categorical data are expressed as numbers, frequencies, or percentages, and were analyzed using the chi-squared test or Fisher's exact test. The time between completion of the surgical procedure and first request for rescue analgesics was plotted as Kaplan–Meier survival curves and compared using the log rank test. *P* < 0.05 was considered significant. Statistical analyses were carried out using SPSS 22.0 (IBM, Armonk, NY, USA).

## Results

### Patient Characteristics at Baseline

**Figure 1:**
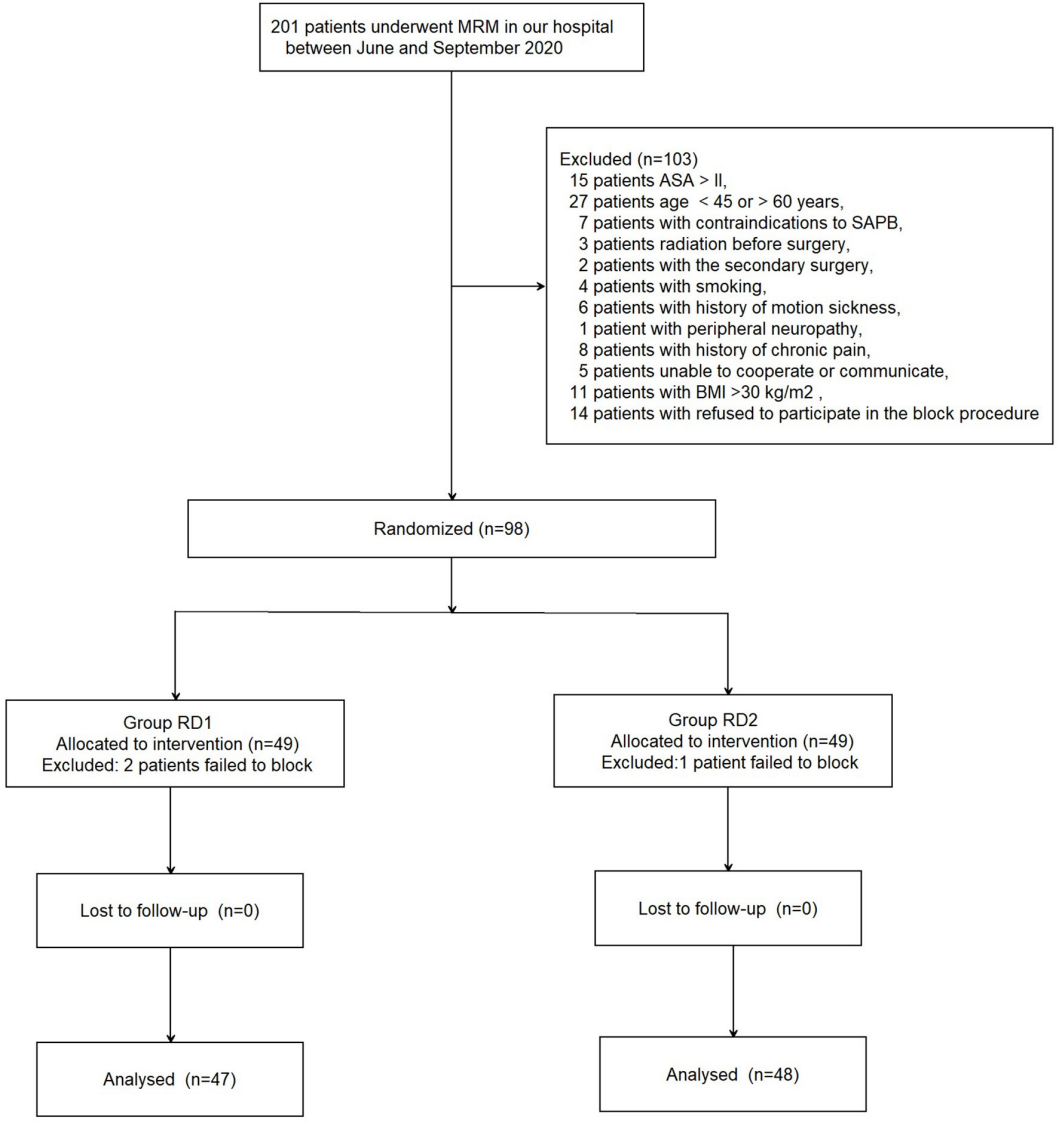
Flowchart showing patient selection.

A total of 201 patients who underwent MRM between June and September 2020 were recruited (see the CONSORT diagram shown as Figure 1). A total of 103 patients were excluded. Ninety-eight patients were included and divided into two groups of 49. In addition, three patients were excluded from analyses because of USG-DSAPB failure (two patients from the RD1 group and one patient from the RD2 group). There were no significant differences between the two groups with respect to demographic data (*P* > 0.05; Table 1).

**Table 1:** Comparison of demographic data between the two groups.

	**Group RD1** **(*n* = 47)**	**Group RD2** **(*n* = 48)**	***P*-values**
Age (years)	52.56 ± 5.83	51.82 ± 6.91	0.572
Body weight (kg)	65.21 ± 6.08	63.89 ± 8.36	0.378
BMI (kg·m^−2^)	22.74 ± 3.01	23.15 ± 3.67	0.551
ASA I/II (*n*)	24/23	30/18	0.261
Comorbidity, *n* (%)	12 (22.53%)	10 (20.83%)	0.965
Hypertension	8 (17.02%)	7 (14.58%)	
Diabetes mellitus	5 (10.64%)	6 (12.50%)	
Coronary heart disease	1 (2.13%)	1 (2.08%)	
COPD/asthma			

*Variables presented as mean ± SD or number of patients n (%). BMI, body mass index; ASA, American Society of Anesthesiology; COPD, chronic obstructive pulmonary disease*.

### Intraoperative Variables

**Figure 2:**
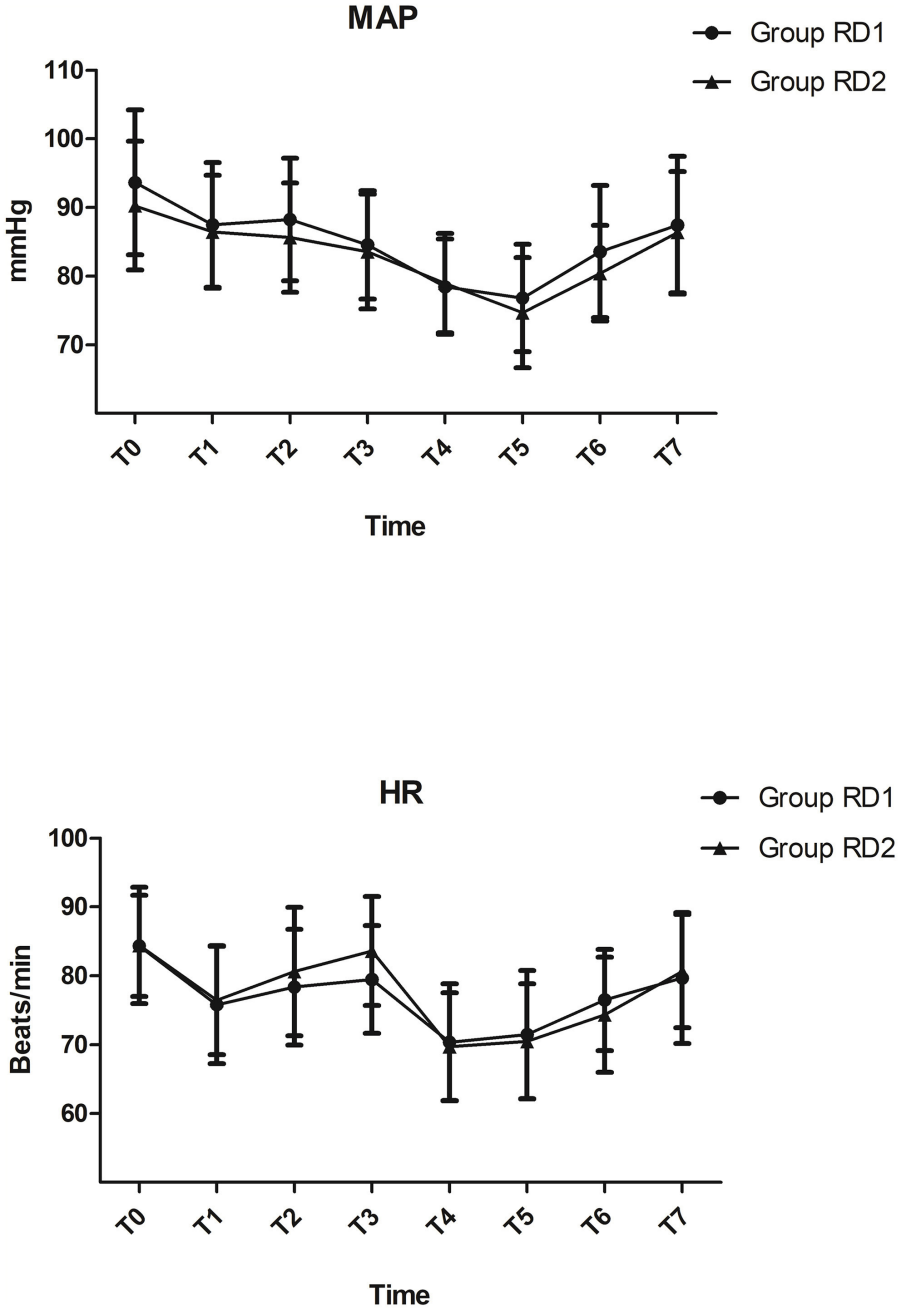
Intraoperative hemodynamics.

MAP and HR were not significantly different between the two groups from t0 to t7 (*P* > 0.05; Figure 2). Consumption of propofol, remifentanil, and DEX, and the number of patients using vasoactive agents were reduced significantly in the RD2 group compared with those in the RD1 group (*P* < 0.05; Table 2). There were no significant differences between the two groups with respect to the duration of the surgical procedure and anesthesia, cisatracurium consumption, one-time puncture success, and procedure time of blockade (*P* > 0.05; Table 2). The duration of PACU stay was significantly shorter in the RD2 group (*P* < 0.05; Table 2). Although the total dermatomal spread was comparable between the two groups [4 (range, 3–5) vs. 4 (range, 3–5) segments; *P* = 0.487], more patients in the RD2 group had T1 and T2 dermatomal spread compared with those in the RD1 group (*P* < 0.05; Table 3).

**Table 2:** Intraoperative data between the two groups.

	**Group RD1** **(*n* = 47)**	**Group RD2** **(*n* = 48)**	***P*-values**
Location (center/right), *n*	30/17	25/23	0.301
Duration of anesthesia (min)	108.83 ± 18.03	115.98 ± 19.38	0.063
Duration of surgery (min)	97.93 ± 10.56	102.36 ± 15.84	0.108
Fluids (ml)	758.82 ± 59.93	808.29 ± 69.08	0.083
Dexmedetomidine (μg·kg^−1^·h^−1^)	0.32 ± 0.06	0.23 ± 0.04^**^	0.001
Remifentanil (μg·kg^−1^·min^−1^)	0.16 ± 0.05	0.10 ± 0.04^**^	0.001
Propofol (μg·ml^−1^)	4.76 ± 1.04	3.52 ± 0.65^*^	0.037
Cisatracurium dosage (mg·kg^−1^)	0.13 ± 0.05	0.14 ± 0.05	0.332
One-time puncture success, *n* (%)	45 (95.74%)	46 (95.83%)	1.000
Block procedure time (min)	5.34 ± 0.98	4.89 ± 0.83	0.268
Number of using vasoactive agent, *n* (%)	12 (25.54%)	7 (14.58%)^*^	0.045
PACU length of stay (min)	23.71 ± 6.92	18.93 ± 4.28^*^	0.018

*Variables presented as mean ± SD or number of patients n (%). PACU, post-anesthesia care unit*.

* *P < 0.05 vs. Group RD1;*

** *P < 0.01 vs. Group RD2*.

**Table 3:** Dermatomal effects after SABP between the two groups.

	**Group RD1** **(*n* = 47)**	**Group RD2** **(*n* = 48)**	***P*-values**
T1	3	10^*^	0.040
T2	14	25^*^	0.027
T3	45	46	1.000
T4	47	48	1.000
T5	43	46	0.435
T6	23	28	0.306
T7	12	17	0.296
T8	4	7	0.355

*Variables presented as number of patients*.

* *P < 0.05 vs. Group RD1*.

### Postoperative Variables

**Figure 3:**
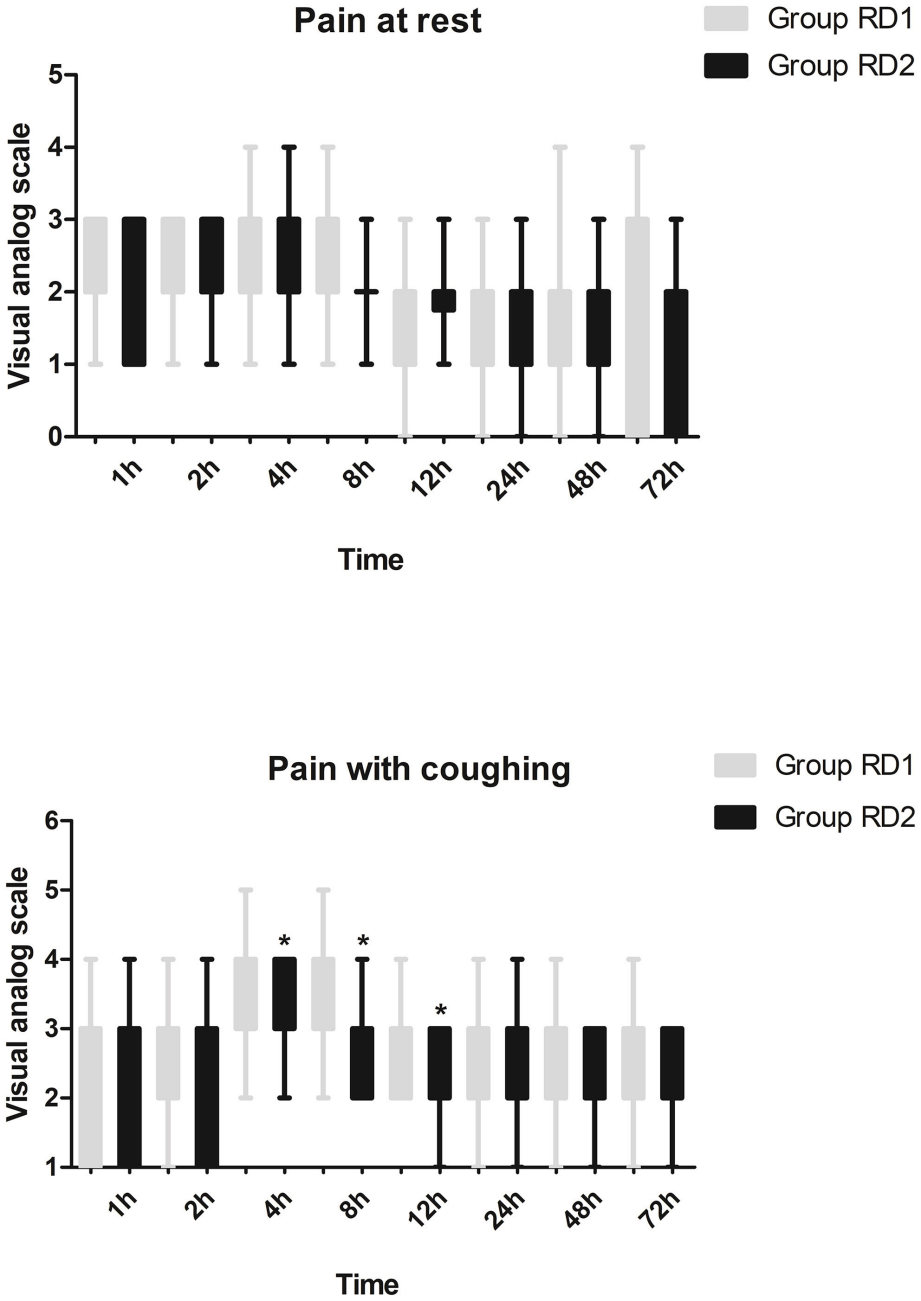
Intensity of postoperative pain between the two groups. *P < 0.05 vs. RD1 group.

**Figure 4:**
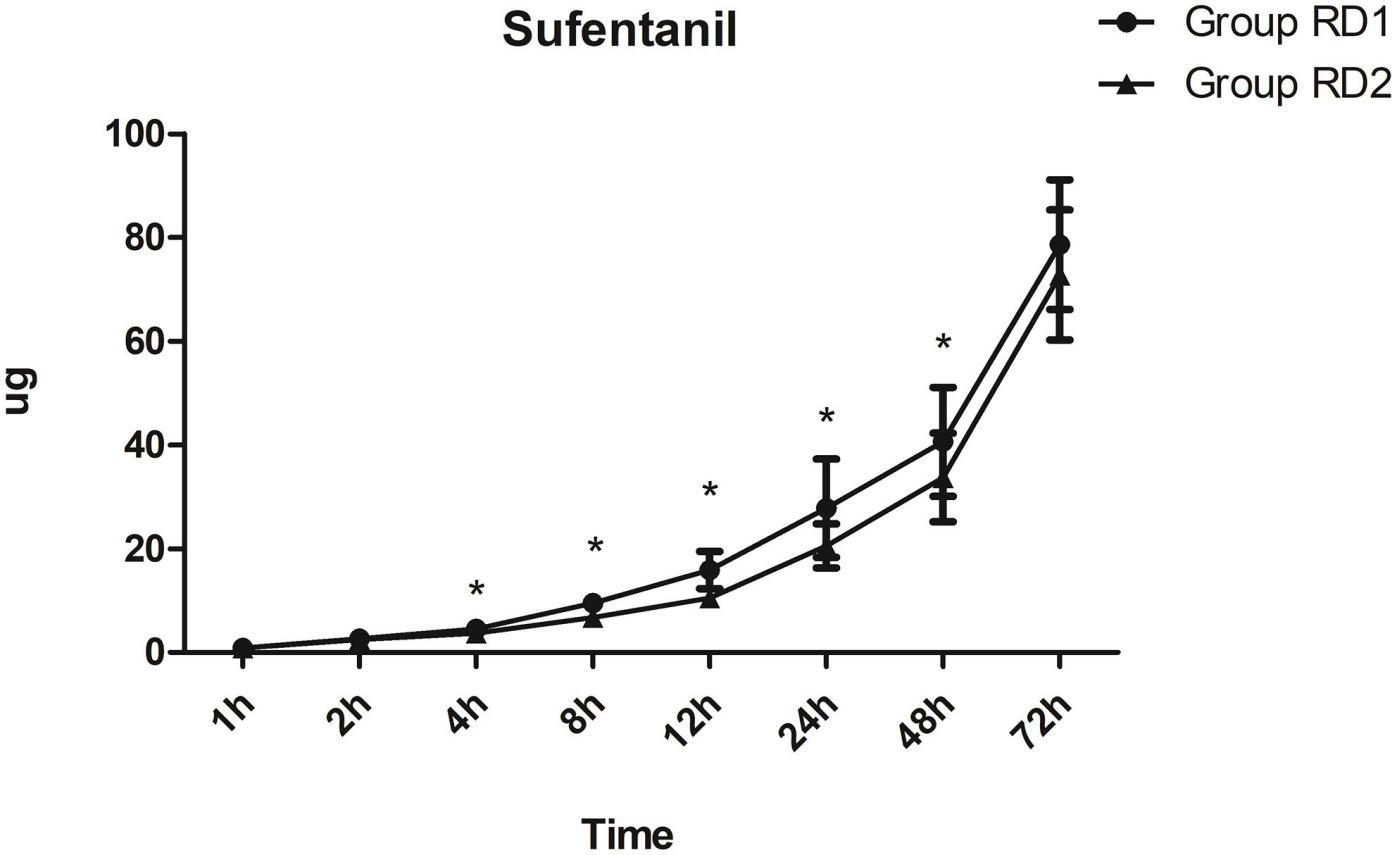
Postoperative sufentanil consumption in the two groups. *P < 0.05 vs. RD1 group.

**Figure 5:**
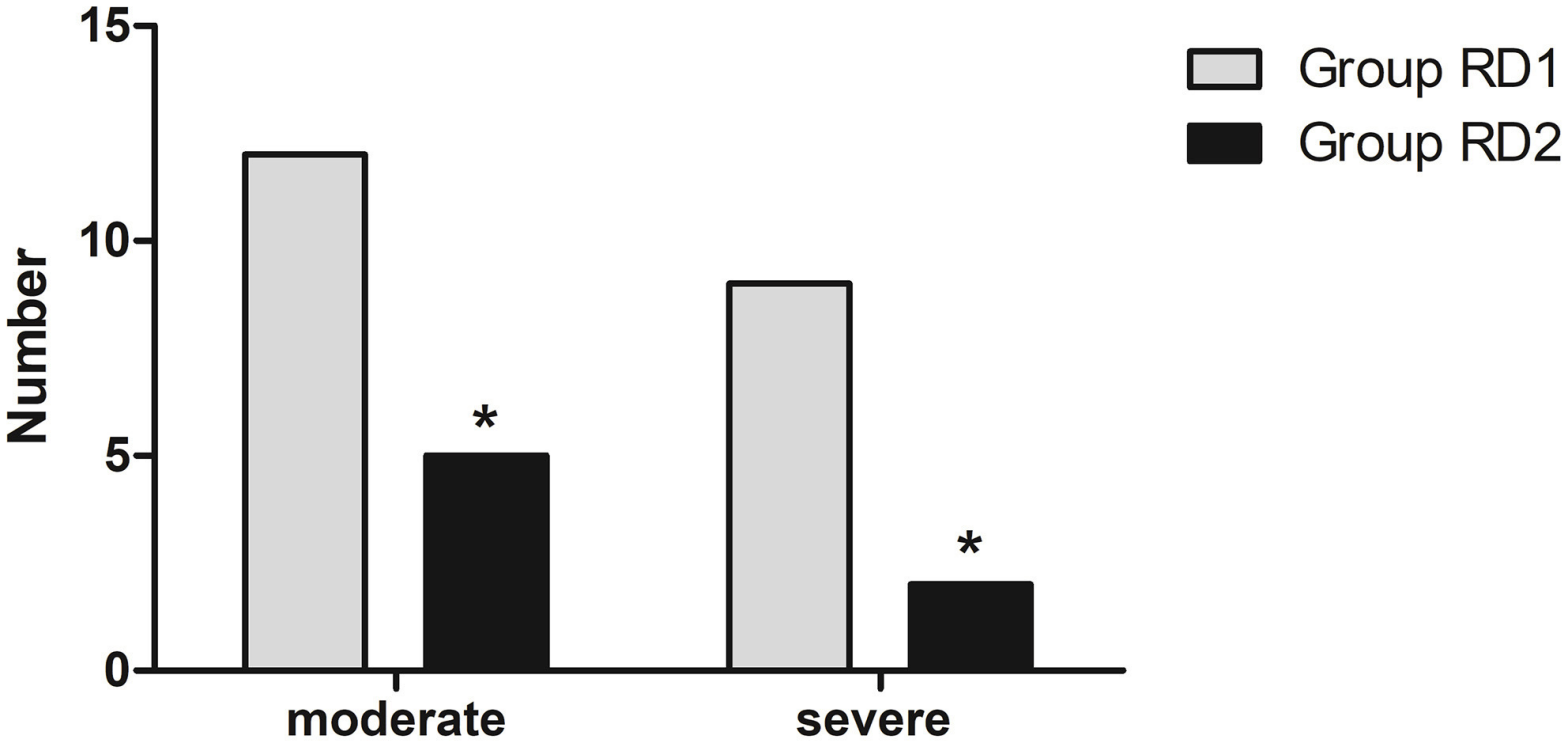
Prevalence of moderate**-**to-severe pain in the two groups. *P < 0.05 vs. RD1 group.

Compared with the RD1 group, only the VAS score for coughing was significantly lower in the RD2 group 4, 8, and 12 h after surgery (*P* < 0.05; Figure 3). Sufentanil consumption was significantly lower in the RD2 group 4, 8, 12, 24, and 48 h after surgery than that in the RD1 group (*P* < 0.05; Figure 4). The prevalence of moderate-to-severe pain was significantly lower in the RD2 group (*P* < 0.05; Figure 5).

**Figure 6:**
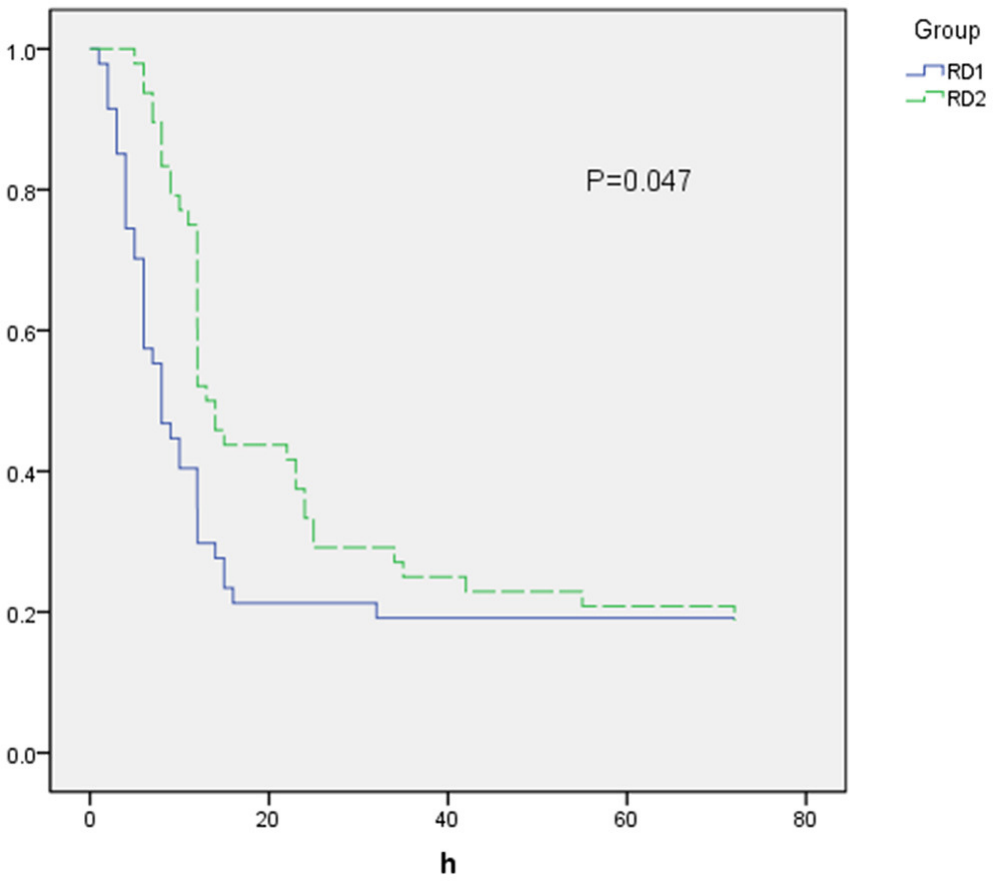
Time to first rescue analgesia.

The time to first rescue analgesia was significantly longer in the RD2 group (*P* = 0.047; Figure 6). The requirement for rescue analgesia was significantly higher in the RD1 group (*P* < 0.05; Table 4). There was no significant difference between the two groups with respect to the level of sedation, or the satisfaction scores for patients and surgeons (*P* > 0.05; Table 4).

**Table 4:** Postoperative level of sedation, rescue analgesia, patients, and surgeons satisfaction between the two groups.

	**Group RD1** **(*n* = 47)**	**Group RD2** **(*n* = 48)**	***P*-values**
**LOS**			0.727
1 h	1 (1, 2)	1 (0–2)	
2 h	1 (0–2)	1 (0–2)	
4 h	1 (0–2)	1 (0–2)	
8 h	0 (0–2)	0 (0–2)	
12 h	0 (0–2)	0 (0–2)	
24 h	0 (0–1)	0 (0–1)	
48 h	0 (0–0)	0 (0–0)	
72 h	0 (0–0)	0 (0–0)	
Patients satisfaction score	4.25 (3.75–5.00)	4.25 (3.75–4.75)	0.093
Surgeons satisfaction score	4.50 (4.25–5.00)	4.50 (4.00–5.00)	0.142

*Variables presented as number of patients n (%) or median (interquartile range). LOS, level of sedation*.

The most common postoperative complication was PONV, which was more frequent in the RD1 group, but the difference was not significant (21.28 vs. 16.67%; *P* = 0.253; Table 5). Cardiovascular-, respiratory-, or blockade-related complications were absent in both groups. The duration of hospital stay, global QoR-40 score, and prevalence of chronic pain were comparable between the two groups (*P* > 0.05; Table 5).

**Table 5:** Comparison of incidence of adverse effects, hospital length of stay, QoR-40, and prevalence of chronic pain between the two groups.

	**Group RD1** **(*n* = 47)**	**Group RD2** **(*n* = 48)**	***P*-values**
**Adverse effects**			
PONV	10 (21.28%)	6 (16.67%)	0.253
Urinary retention	5 (10.64%)	5 (10.42%)	0.972
Itching	4 (8.51%)	3 (6.25%)	0.714
Cardiovascular complications	0	0	1.000
Respiratory complications Block related	0	0	1.000
complications	0	0	1.000
Hospital length of stay	6 (4–8)	5 (5–7)	0.302
Prevalence of chronic pain, n (%)	15 (31.91%)	13 (27.08%)	0.606
**Global QoR-40**			
Pre-operative	185 (178–197)	186 (174–195)	0.521
Postoperative day 1	167 (153–186)	172 (158–188)	0.096
On discharge	177 (174–188)	179 (174–186)	0.277
3 month later	182 (166–192)	184 (164–195)	0.193

*Variables presented as number of patients n (%) or median (interquartile range). PONV, postoperative nausea and vomiting; QoR-40, 40-item Quality of Recovery questionnaire*.

## Discussion

We showed that 1 μg·kg^−1^ (not 0.5 μg·kg^−1^) of DEX combined with 20 mL of 0.5% ropivacaine and 5 mg of dexamethasone in USG-DSAPB could provide superior postoperative analgesia for patients undergoing MRM. The prevalence of moderate-to-severe pain, number of patients using vasoactive agents, and duration of PACU stay were all significantly lower, whereas the time to first rescue analgesia was significantly longer, in the RD2 group.

Regional anesthesia has gained popularity for its opioid-sparing strategies. High consumption of opioids can lead to greater pain sensitization and increases the risk of developing sensory disturbances following surgery (19, 25–27). Interpectoral plane blockade and pectoserratus plane blockade can be employed to obtain blockade of the upper intercostal nerves as well as reduce resting and dynamic pain scores and morphine-equivalent consumption. However, such blockade should be avoided because of concerns regarding the disruption of tissue within the axilla and need for more than one injection (28–31).

USG-DSAPB has been proposed to provide analgesia to the hemithorax. Scholars have reported that the analgesic effect of DSAPB is influenced by: patient position; the volume, concentration, and physicochemical characteristics of local anesthetic; local tissue conditions; the site and rapidity of injection; use of adjuvants. Of these, the volume may be the critical factor influencing the extent of injectate spread (32).

Compared with other long-acting local anesthetics (e.g., bupivacaine, levobupivacaine), ropivacaine has been used widely in regional anesthesia because of its larger maximum dose and lower systemic toxicity and neurotoxicity (33). Kunigo et al. reported that SAPB with 40 mL of 0.375% ropivacaine diffused to a greater extent than 20 mL of 0.375% ropivacaine. However, there was no significant difference between the two groups with the respect to the time to analgesic rescue or impact on posterior spread. Hence, 20 mL of ropivacaine could be safer and help avoid local toxicity (20, 34). Researchers have reported that comparison of use of 0.5 and 0.75% ropivacaine revealed no significant difference upon postoperative analgesia, but that both were superior to use of 0.375% ropivacaine. Those data indicated that an increase in the ropivacaine concentration may be a better approach to improve the analgesic efficacy of DSAPB and prolong the duration of pain relief (35, 36). Hence, we adopted DSAPB with 0.5% ropivacaine for preemptive analgesia. Several reports have shown that low concentrations of ropivacaine can be detected in blood, which may result in sedation by suppressing functioning of sodium channels in cells in the central nervous system (37, 38). Therefore, we recorded the level of sedation 72 h after surgery (though the results were similar between the two groups).

Deep blockade does not disrupt the surgical-tissue planes or spare blockade of the long thoracic nerve (which would occur in superficial blockade) and preserved scapula function (39). Besides, deep blockade can avoid the injection of local anesthetic into the plane during lymph-node dissection or axillary clearance and promote greater caudad spread of local anesthetic (40). Thus, we adopted USG-DSAPB even though it poses a higher risk of pneumothorax (41). We defined “successful blockade” as the loss of cold sensation in >2 dermatomes before surgery instead of after surgery to reduce the risk of a recall bias. We used USG-DSAPB as preemptive analgesia before surgery, and it might be more effective in reducing hyperalgesia, allodynia, central sensitization, and provide a better risk:benefit ratio (42). In addition, DEX probably produces peripheral analgesic effects by inhibiting the transmission of nerve signals through A-delta fibers and C-fibers and stimulates the release of enkephalin-like substances in peripheral regions (21, 43). Studies have also reported that use of corticosteroids as adjuvants can prolong analgesia significantly, reduce postoperative pain, and increase glycemia only slightly on postoperative day-1 (44). As a result, the duration of postoperative analgesia of USG-DSAPB in our study was longer than that reported previously. Consistent with the results of a study by Kamiya et al. (45), we also recorded rebound pain 24 h after surgery. The younger female patients recruited in our study may have felt more intense pain after the analgesic effect of DSAPB had disappeared, and an acute state of opioid-induced tolerance and hyperalgesia occurred.

The number of patients who required vasoactive agents intraoperatively was significantly higher in the RD1 group. The reason may be due to the efficacy of a higher dose of DEX in USG-DSAPB in attenuating the sympathetic response to surgical stimulation. However, according to De Cassai et al. (46), the risk of bradycardia and hypotension should not be ignored, and vasoactive agents should be used cautiously. The time to first rescue analgesia was significantly longer and the requirement for rescue analgesia was significantly higher in the RD2 group. However, there was no significant difference between the two groups with respect to the satisfaction scores of patients and surgeons. Though 10 mL of 2% lidocaine was infiltrated subcutaneously in parasternal and subclavicular areas upon completion of surgery, most of the postoperative requirements for rescue analgesia were due to incomplete analgesia in the internal mammary area. Recently, ultrasound-guided transversus thoracic muscle plane blockade or parasternal intercostal blockade have been introduced to provide analgesia in the internal mammary area, which cannot be blocked completely by DSAPB (47, 48).

No complications were associated with USG-DSABP, likely because the target point of SAPB is superficial and we adopted ultrasound-guided real-time SAPB to prevent misplacement of the needle tip (49). Consistent with the result of a recent meta-analysis of randomized controlled trials, few patients in our study suffered from PONV, likely due to prophylactic administration of antiemetics before surgery completion and low consumption of opioids (40). Besides, O'Scanaill et al. (28) reported that DEX reduced the prevalence of PONV by reducing movement of the stomach and intestines, inhibiting glandular secretion, and reducing the opioid dose. In opposition with the results of a study by Wang L and collaborators, we did not record differences between the two groups with respect to chronic pain even though opioid consumption was lower in the RD2 group. The reason may be because we recruited patients only undergoing dissection of axillary lymph nodes, which is an independent risk factor for chronic pain (50).

Our study had three main limitations. First, for ethical considerations, we could not create a sham group injected with placebo instead of local anesthetic. Second, we did not measure the plasma concentration of ropivacaine for economic reasons. Third, we recruited patients only with ASA grade I–II; discussed special types of patients such as patients with obstructive sleep apnea maybe more meaningful.

## Conclusions

We discovered that 1 μg·kg^−1^ (not 0.5 μg·kg^−1^) DEX combined with 20 mL of 0.5% ropivacaine and 5 mg of dexamethasone in USG-DSAPB could provide superior postoperative analgesia for patients undergoing MRM. The prevalence of moderate-to-severe pain, the number of patients needing vasoactive agents, and duration of PACU stay were significantly lower, whereas the time to first rescue analgesia was significantly longer, for patients undergoing MRM with 1 μg·kg^−1^ DEX in USG-DSAPB. Well-designed and appropriately powered randomized controlled trials are needed to explore the optimal dose of DEX for USG-DSAPB.

## Data Availability Statement

The raw data supporting the conclusions of this article will be made available by the authors, without undue reservation.

## Ethics Statement

Ethical approval was obtained from the Institutional Review Boards of both Liaocheng People's Hospital and Ordos Central Hospital, and then registered at Chinese Clinical Trial Registry (ChiCTR2000033685). The patients/participants provided their written informed consent to participate in this study. Written informed consent was obtained from the individual(s) for the publication of any potentially identifiable images or data included in this article.

## Author Contributions

XX, XC, and YQ conceived and designed the study. WZ analyzed the data. XC, JZ, YL, and CD collected the data. XX, XC, WZ, CD, and YQ wrote the manuscript. All authors contributed to the article and approved the submitted version.

## Conflict of Interest

The authors declare that the research was conducted in the absence of any commercial or financial relationships that could be construed as a potential conflict of interest.

## Publisher's Note

All claims expressed in this article are solely those of the authors and do not necessarily represent those of their affiliated organizations, or those of the publisher, the editors and the reviewers. Any product that may be evaluated in this article, or claim that may be made by its manufacturer, is not guaranteed or endorsed by the publisher.
